# Proteomic Profiling for Identification of Novel Biomarkers Differentially Expressed in Human Ovaries from Polycystic Ovary Syndrome Patients

**DOI:** 10.1371/journal.pone.0164538

**Published:** 2016-11-15

**Authors:** Li Li, Jiangyu Zhang, Qingshan Deng, Jieming Li, Zhengfen Li, Yao Xiao, Shuiwang Hu, Tiantian Li, Qiuxiao Tan, Xiaofang Li, Bingshu Luo, Hui Mo

**Affiliations:** 1 Department of Obstetrics and Gynecology, Guangdong Women and Children Hospital, Guangzhou, 510010, China; 2 Department of Pathology, Guangdong Women and Children Hospital, Guangzhou, 510010, China; 3 The Third Affiliated Hospital of Southern Medical University, Guangzhou, 510515, China; 4 Laboratory of Chinese Medicine Quality Research, Macau University of Science and Technology, Macau, 00853, China; 5 Department of Pathophysiology, Southern Medical University, Guangzhou, 510515, China; John Hopkins University School of Medicine, UNITED STATES

## Abstract

**Objectives:**

To identify differential protein expression pattern associated with polycystic ovary syndrome (PCOS).

**Methods:**

Twenty women were recruited for the study, ten with PCOS as a test group and ten without PCOS as a control group. Differential in-gel electrophoresis (DIGE) analysis and mass spectroscopy were employed to identify proteins that were differentially expressed between the PCOS and normal ovaries. The differentially expressed proteins were further validated by western blot (WB) and immunohistochemistry (IHC).

**Results:**

DIGE analysis revealed eighteen differentially expressed proteins in the PCOS ovaries of which thirteen were upregulated, and five downregulated. WB and IHC confirmed the differential expression of membrane-associated progesterone receptor component 1 (PGRMC1), retinol-binding protein 1 (RBP1), heat shock protein 90B1, calmodulin 1, annexin A6, and tropomyosin 2. Also, WB analysis revealed significantly (P<0.05) higher expression of PGRMC1 and RBP1 in PCOS ovaries as compared to the normal ovaries. The differential expression of the proteins was also validated by IHC.

**Conclusions:**

The present study identified novel differentially expressed proteins in the ovarian tissues of women with PCOS that can serve as potential biomarkers for the diagnosis and development of novel therapeutics for the treatment of PCOS using molecular interventions.

## Introduction

Polycystic ovary syndrome (PCOS) is a hyperandrogenic disorder associated with chronic oligoanovulation and polycystic ovarian morphology that affects 6–10% of the reproductive women [1, 2]. The prevalence of PCOS in the Chinese population is reportedly 5.6% [3]. According to the Rotterdam diagnostic criteria, the prevalence of PCOS may reach 15–20% [4]. A PCOS diagnosis may be associated with menstrual problems in adolescence [5], reduced fertility due to an ovulatory disorder, and an increased predisposition to miscarriage and other pregnancy-related complications (gestational diabetes, preterm delivery, and pre-eclampsia) [6]. In addition to the symptoms of androgen excess and reproductive consequences, PCOS is associated with long-term risk for the development of severe metabolic disorders including obesity, diabetes, and cardiovascular disease [7, 8]. A recent systematic review and meta-analysis showed that women with PCOS were four times more likely to develop type 2 diabetes mellitus (T2DM) compared with the body mass index (BMI)-matched controls [9]. A major retrospective study revealed that patients with PCOS suffered from an increased risk of cardiovascular diseases, metabolic diseases, psychological diseases, tumors, and reproductive abnormalities [10]. Some reports indicated that women with PCOS were at a markedly increased risk of endometrial cancer (relative risk = 2.7; 95% confidence interval, 1.0–7.29) [11], a finding that was confirmed by the subsequent systematic review that revealed a three-fold increased risk [12].

The pathogenesis of PCOS is complex, and its etiology remains unclear. Thus, a greater understanding of its heterogeneous etiology should lead to improved therapeutic intervention. Experiments with ovarian theca cells demonstrated that excess androgen was a primary defect in women with PCOS [13]. Moreover, approximately 50–70% of patients with PCOS have insulin resistance (IR) and compensatory hyperinsulinism [14] proposed key pathophysiological features of PCOS that contribute to reproductive, cardiovascular, and metabolic disturbances. It is suggested that women with PCOS often have more severe pathologically distinct insulin resistance than those in weight-matched non-PCOS populations [15].

Non-targeted proteomics has been used in the past few years with the aim of identifying molecules potentially involved in the pathophysiology of PCOS. Such approaches can link changes in the protein function with comprehensive changes in protein expression and posttranslational modification and hold great promise for unraveling the mechanism of disease, providing new insights about PCOS. The integration of the huge amount of novel information obtained from proteomics may contribute to the elucidation of cellular modifications, resulting in PCOS. A large number of biomolecules involved in metabolism, androgen biosynthesis, or chronic inflammation pathways have been studied in previous reports [16–19]. These proteins provide vital information about altered molecular functions in PCOS and raise questions concerning their precise role in its pathogenesis. Pinpointing such molecules may even lead to the development of specific diagnostic techniques and the identification of new therapeutic targets. Here, we planned to employ a proteomics-based approach to identify proteins associated with PCOS, and provide a framework for a systemic approach for profiling the biomarkers in the future.

## Materials and Methods

### Subject selection

The process was approved by the Ethics Committee of Guangdong Province Maternal and Children Health Hospital. All the subjects, undergone surgery in 2013–2014, were recruited from Guangdong Province Maternal and Children Hospital, China. Written informed consent was obtained from all the participants.

During the selection of patients with PCOS, the diagnosis relied on the combination of clinical symptoms, ultrasonographic examination, and biochemical data as per guidelines of the revised diagnostic criteria announced in the 2003 American Society for Reproductive Medicine/European Society of Human Reproduction and Embryology Rotterdam consensus [20]. The PCOS patients presented with at least two of the three following criteria: oligomenorrhea or amenorrhea, hyperandrogenism or clinical signs of hyperandrogenism (hirsutism or acne), and polycystic ovaries in ultrasound. The patients were selected because they were undergoing ovarian wedge resection: as a second line therapy for PCOS after they failed to respond to Clomiphene treatment. Therefore, the number of patients undergoing ovarian wedge resection during the study period determined the sample size. Hence, the test group consisted of ten PCOS patients. Consequently, the same number (ten) of patients with benign ovarian teratoma or ovarian cysts that underwent ovary resection was enrolled as the control group.

For both test and control group, participants suffering from diseases, such as adrenal hyperplasia, Cushing's syndrome, androgen-secreting tumors, thyroid disease, hyperprolactinemia, severe medical illness, and mental disorders, were excluded from the study.

### Sample collection

Surgical wedge resection of the ovaries was performed under laparoscopy in all the patients with PCOS. The volume of the wedge biopsy was approximately one-tenth of the overall ovary volume. Ovarian biopsies were randomly collected because these patients did not have a regular menstrual cycle. Histological evaluation of two samples revealed multiple small subcortical follicles and increased stromal hyperplasia characteristic of PCOS.

The normal ovary samples in the control group were taken from the biopsy of one ovary in patients whose other ovary was resected to treat a unilateral benign ovarian teratoma or an ovarian cyst. All the samples were collected during the follicular phase of the menstrual cycle. Histological evaluation revealed the presence of developing follicles and the ovarian stromal compartment of each ovary appeared normal.

The ovarian biopsies constituted of minimal wedges taken at the equatorial plane at the edge of each ovary. Care was taken to ensure that both cortical and stromal components were collected. The biopsies constituted approximately one-tenth of each ovary and had a pyramidal shape with the top of the pyramid located at the ovarian hilus. The specimens were collected immediately after the surgery; a randomly selected quarter was snap frozen in liquid nitrogen, and stored at -80°C until further analysis, while the remaining tissue samples were subjected to immunohistochemistry (IHC).

### Sample preparation

Tissue proteins were extracted and digested as described by Wang et al. [21]. Frozen tissue samples were weighed (100 mg/mL lysis buffer) and thawed on ice. The samples were resuspended in lysis buffer (30 mM Tris-HCl, 7 M urea, 2 M thiourea, 4% CHAPS; pH 8.5) and incubated on ice for 30 min. The suspensions were sonicated on ice for five cycles consisting of 10 sec bursts with 30 sec pause. The lysates were then centrifuged at 12,000 *g* for 30 min. The solubilized proteins were precipitated with a 2-D Clean-Up Kit (GE Healthcare BioSciences, Little Chalfont, UK) according to the instructions of the manufacturer and resuspended in the lysis buffer. The protein concentration was determined using a 2-D Quant Kit (Amersham Biosciences, USA)- Aliquots of the protein samples were frozen or freeze-dried. All reagents were procured from Sigma unless otherwise noted.

### Differential in-gel electrophoresis (DIGE)

Three randomly selected ovarian tissue samples were pooled, each from the test and the control group. For DIGE, 50 μg of the protein was minimally labeled with CyDyes at the ratio of 1 μg protein:400 pmol Cy3 or Cy5 protein-labeling dye (GE Healthcare). Cy3 and Cy5 were used to label the samples, and Cy2 was used to label the internal standard (a pool of samples) (S1 Table). The labeled samples were applied to a 24-cm immobilized pH gradient gel (IPG) strip (pH 3–10 NL), and the first-dimension isoelectric focusing (IEF) was performed at 20°C in IPGphor III (GE Healthcare). After the IEF, the strips were loaded onto a second-dimensional 24 × 24 cm 12% polyacrylamide gel slab, and separation performed on the DALTsix System (GE Healthcare). The gels were scanned on a Typhoon 9400 imager (GE Healthcare) and analyzed with DeCyder 2D Software V6.5 (GE Healthcare). Filtering conditions of at least 50% change of ratios between both the groups was applied to select the protein spots that were differentially expressed between the groups. For picking the differentially expressed spots, a similar gel loaded with 600 μg unlabeled pooled protein sample was used and stained with colloidal Coomassie Blue G-250. An Ettan Spot Picker (GE Healthcare) was employed to pick the matched spots.

### Matrix-assisted laser desorption ionization/time-of-flight MS analysis and protein identification

The differentially expressed spots were excised from the Coomassie Blue-stained gel and destained with 50% acetonitrile (ACN)/100 mM NH_4_HCO_3_ for 10 min. Subsequently, each gel piece was subjected to trypsin (25 ng/mL in NH_4_HCO_3_) treatment in 30 μL of 50 mM NH_4_HCO_3_. After overnight incubation at 37°C, the peptide mixtures from the gel pieces were extracted, dried using a vacuum pump, and solubilized in 2 μL of 50% ACN/0.1% trifluoroacetic acid (TFA). It was then dehydrated with 0.5 μL of matrix solution containing alpha-cyano-4-hydroxycinnamic acid saturated in 50% ACN/0.1% TFA. The samples were assessed by an ABI 4800 Proteomics Analyzer matrix-assisted laser desorption/ionization time-of-flight (MALDI-TOF) mass spectrometer (MS; Applied Biosystems). For the MS analyses, 800 spots were typically accumulated. MS/MS analyses were performed at a collision energy of 2 KV with air. The Mascot search engine (version 2.1; Matrix Science) and GPS Explorer™ software version 3.6.2 (Applied Biosystems) were used to explore the tandem mass spectra of the peptide and protein. The Mascot search engine was used to identify the proteins.

### Western Blot

For the validation of the differentially expressed proteins, ten PCOS and ten normal ovarian tissue samples were subjected to western blot (WB) analysis. Protein concentrations of the lysates were estimated by the bicinchoninic acid protein quantification method. 50 μg protein was separated by polyacrylamide gel electrophoresis and transferred to nitrocellulose membrane. The membrane was blocked for 1 h at room temperature and incubated with primary anti-membrane-associated progesterone receptor component 1 (PGRMC1) rabbit polyclonal antibody (1:900; Abcam), anti- retinol-binding protein 1 (RBP1) mouse antibody (1:2000; Abcam), heat shock protein 90B1 (HSP90B1, 1:200; Cell Signaling Technology, China), calmodulin 1 (CALM1), annexin A6 (ANXA6), and tropomyosin 2 (TPM2) (1:200; Abcam) overnight at 4°C. Antibody against glyceraldehyde-3-phosphate dehydrogenase (GAPDH) was used for the detection of an internal control protein, GAPDH. The blot was washed twice and incubated with the HRP-conjugated secondary antibody (R&D) for 1 h. The blots were developed with ECL (Pierce) and scanned using ImageQuant LAS 4000 biomolecular imager with CCD camera system (Springer Lab) and the signal intensity of each band was quantified using Image J software. Relative protein levels of the test and control samples were normalized to GAPDH.

### Immunohistochemistry (IHC)

Ten PCOS and ten normal ovarian tissue samples were selected for IHC analysis. Ovarian tissues were embedded in optimal cutting temperature medium for sectioning. The sections (7 μm each) were fixed with 4% paraformaldehyde and permeabilized with 1% Triton X-100. Subsequently, the sections were washed twice with phosphate-buffered saline (PBS) and incubated with antibodies against S phase–related PCNA (1:500; Santa Cruz Biotechnology), PGRMC1 (1:900; Abcam), RBP1 (1:200; Abcam), HSP90 (1:200; Cell Signaling Technology), CALM1, ANXA6, and TPM2 (1:200; Abcam) for 2 h. The sections were washed twice with PBS and incubated with secondary horseradish peroxidase–conjugated antibodies for 1 h. Finally, the sections were washed thrice with PBS and developed using 3,3-diaminobenzidine (DAB). The positively stained cells were visualized under a contrast light microscope. Images were captured using an Eclipse TE 2000-U fluorescence microscope (Nikon, Japan). The protein expression was analyzed quantitatively by cell counting, and the data presented as the mean ratio of the cell counts of positive cells to the nuclei ± standard error (SE).

### Statistical analysis

A two-tailed unpaired Student’s t-test was employed to compare the test and control group (mean ± SD). The DeCyder software was used for the statistical evaluation of the DIGE gels. For all the protein spots, a comparison between PCOS and control groups was calculated, as a change in volume ratios. To identify statistically confident differentially expressed protein spots, a filtering condition of at least 50% change in the ratio between the test and control was set. For WB and IHC, two-tailed non-paired Student’s t-test was used to determine the mean differences between the two groups. A P<0.05 was used to assess the significance of the difference using SPSS 17.0 software.

## Results

### Patients

To identify the proteins differentially expressed in PCOS, ovary samples from ten PCOS and ten control patients were collected during the period 2013–2014. The PCOS patient group consisted of ten women with a mean age of 28.60 years (range, 22–33 years), mean BMI of 27.21 kg/m^2^ (range, 22.7–33.5 kg/m^2^), and mean menstrual cycle duration of 103.10 days (range, 63–200 days). Serum luteinizing hormone levels were elevated (mean, 10.27 IU/L; range, 4.34–21.2 IU/L), whereas follicle stimulating hormone concentrations were within the normal range (mean, 5.64 IU/L; range, 3.71–8.13 IU/L). The mean testosterone level was elevated at 2.21 nmol/L (range, 1.43–3.70 nmol/L), and the mean sex hormone binding globulin level was 81.71 nmol/L (range, 19.8–363 nmol/L). The mean ovary volumes of PCOS patients were 10.03 ± 1.09 (left) and 11.54 ± 0.98 (right) mm^3^ (Table 1). All patients had a long-standing history of infertility, and all underwent ovulation induction without achieving pregnancy.

**Table 1:** Clinical data of the selected subjects.

	PCOS (n = 10)	Control (n = 10)	P value
**Age (years)**	28.60 ± 1.04	28.60 ± 1.38	1.0000
**BMI (kg/m**^**2**^**)**	27.21 ± 0.90	25.87 ± 1.07	0.3498
**Menstrual cycle duration (days)**	103.10 ± 16.93	27.40 ± 1.176	0.0003
**FSH (IU/L)**	5.64 ± 0.44	6.24±1.11	0.1295
**LH (IU/L)**	10.27 ± 1.72	5.39±1.52	<0.0001
**SHBG (nmol/L)**	81.71 ± 33.93	89.21±24.15	0.5761
**T (nmol/L)**	2.21 ± 0.24	1.37±0.21	<0.0001
**Ovary (mm**^**3**^**)**			
**Left**	10.03 ± 1.09	8.03 ± 1.21	0.0011
**Right**	11.54 ± 0.98	8.74 ± 0.56	<0.0001

PCOS, polycystic ovary syndrome; BMI, body weight index; FSH, follicle-stimulating hormone; LH, luteinizing hormone; SHBG, sex hormone binding globulin; T, testosterone.

The control group consisted of ten women with a median age of 28.6 years (range, 22–34 years), median BMI of 25.9 kg/m^2^ (range, 20.7–30.5 kg/m^2^), and with a relative menstrual cycle duration (mean, 27.4 days; range, 24–35 days) that was shorter than the PCOS group (P = 0.0003) (Table 1). The ovary resection information of control group is summarized in S2 Table.

### MALDI-TOF MS analysis

The ovarian tissues from patients with PCOS and control were analyzed for differential protein expression by 2D-DIGE analyses. Statistically confident (P<0.05) differentially expressed protein spots were identified by applying a filtering condition of at least 50% change in the ratio between the test and control. A representative scan of a three dye-scanned overlay image is depicted in S1 Fig.

A total of 18 differentially expressed proteins were identified. The MS identification data is presented in Table 2 and S2 Fig. Thirteen proteins were upregulated in PCOS ovaries, whereas five proteins were downregulated. The upregulated proteins were identified as heat shock protein 90 (HSP90B1), retinol-binding protein 1 (RBP1), membrane-associated progesterone receptor component 1 (PGRMC1), calmodulin 1 (CALM1), protein SET (SET), 14-3-3 protein epsilon (YWHAE), calreticulin (CALR), tubulin-β chain (TUBB), polymerase I and transcript release factor (PTRF), complement component 1 Q subcomponent-binding protein (C1QBP), vimentin (VIM), neutral alpha-glucosidase AB (GANAB), and antithrombin III (SERPINC1); and the down-regulated proteins were tropomyosin beta chain (TPM2), annexin A6 (ANXA6), 78 kDa glucose-regulated protein (HSPA5), prelamin-A/C (LMNA), and serum albumin (ALB).

**Table 2:** Significantly (P<0.05) differentially expressed PCOS proteins.

Spot No.	Protein name	Accession No.	MW (kDa)	PI	MOWSE Score	Number of unique peptides	P value	Fold of change (PCOS/Ctrl)
2247	Calmodulin 1	P62158	16.8	4.09	62	3	3.4e-005	5.09
1480	Protein SET	Q01105	33.5	4.23	54	2	2.9e-005	2.19
1713	14-3-3 protein epsilon	P62258	29.3	4.63	71	2	3.9e-005	1.94
877	Heat shock protein 90B1	P14625	92.7	4.76	96	3	5.2e-005	1.84
1241	Calreticulin	P27797	48.3	4.29	83	5	4.5e-005	1.75
1248	Tubulin-β chain	P07437	50.1	4.78	68	4	3.7e-005	1.72
1659, 1680	Polymerase I and transcript release factor	Q6NZI2	43.4	5.51	56	2	3.0e-005	1.56, 1.65
1666	Complement component 1 Q subcomponent-binding protein	Q07021	31.7	4.74	74	2	4.0e-005	1.63
2026	Vimentin	P08670	53.7	5.06	89	3	4.8e-005	1.63
833	Neutral alpha-glucosidase AB	Q14697	107	5.74	72	3	3.9e-005	1.53
2280, 2285	Retinol-binding protein 1	P09455	16.01	4.99	90, 82	3, 2	4.8e-005, 4.4e-005	1.53, 1.52
1931	Membrane-associated progesterone receptor component 1	O00264	21771.8	4.56	86	5	4.9e-005	1.51
1155	Antithrombin-III	P01008	53.03	6.32	65	3	3.5e-005	1.5
1057	78 kDa glucose-regulated protein	P11021	72.4	5.07	81	4	4.4e-005	-1.52
1265	Prelamin-A/C	P02545	74.4	6.57	58	3	3.1e-005	-1.55
1540	Tropomyosin beta chain	P07951	32.9	4.66	63	4	3.4e-005	-1.58
998, 1001, 1009, 1026, 1036, 1040, 1043, 1046, 1074, 1075	Serum albumin	P02768	71.3	5.92	80,72,95,64,71, 54, 59, 48, 75, 82	5, 3, 2, 3, 3, 4, 2, 4, 2, 3	4.3e-005, 3.9e-005, 5.2e-005, 3.5e-005, 3.9e-005, 2.9e-005, 3.2e-005, 2.6e-005, 4.1e-005, 4.5e-005	-1.63, -1.58, -1.54, -1.68, -1.72, -1.60, -1.63, -1.71, -1.70, -1.55
1030	Annexin A6	P08133	76.2	5.42	86	3	4.7e-005	-1.77

### WB and IHC analysis

To further validate the differentially expressed PCOS proteins identified from DIGE, the expression levels of ANXA 6, TPM2, HSP90B1, CALM1, RBP1, and PGRMC1 in ovary tissues of PCOS patients (n = 10) and controls (n = 10) were compared using WB and IHC. A representative WB for all the twenty analyzed samples is shown in S3A Fig. The WB analysis from each of the PCOS (n = 10) and control patients (n = 10) exhibited a significant (P<0.01) increase in the expression of PGRMC1, RBP1, HSP90B1, and CALM1 and a significant (P<0.05) decrease in the expression of ANXA6 and TPM2 in the ovary of PCOS in comparison to the control patients (S3B Fig). The IHC analysis of PCOS (n = 10) and control (n = 10) demonstrated similar effects in the expression of PCOS proteins. A representative IHC image for all the twenty analyzed samples is shown in S4A Fig. The IHC analysis showed a significant increase (P<0.01) in the expression of HSP90B1, CALM1, RBP1, and TPM2 and significant decrease (P<0.05) in the expression of PGRMC1 and ANXA6 (S4B Fig).

## Discussion

Identifying differentially expressed protein profiles in the ovarian tissues of patients with PCOS has important implications for the development of novel therapeutics. The present study identified eighteen proteins of which thirteen are novel, which were differentially expressed between PCOS and normal ovaries. Thirteen proteins were upregulated, and 5 were downregulated in PCOS ovaries. The identified biomolecules might contribute to a better understanding of the pathophysiology, develop novel therapeutics, and estimate long-term health risks of PCOS.

The identified differentially expressed proteins in PCOS ovaries are involved in several pathways, including the regulation of cellular physiological processes and metabolism, fibrinolysis and thrombosis, immune response, insulin resistance, and low-grade chronic inflammation. For example, cellular physiological processes including cell homeostasis and proliferation (such as protein HSP90B1, CALM1, and PGRMC1), regulation of fibrinolysis and thrombosis (such as protein antithrombin-III, SERPINC1), and low-grade chronic inflammation (such as complement component 1 Q subcomponent-binding protein, mitochondrial, CIQBP).

Many different proteomic approaches have found various differentially expressed proteins in women with PCOS with respect to controls [22]. These include genes involved in the insulin signaling pathway [23], proteins associated with cell proliferation [24], and estrogen and progesterone receptor subtypes [25]. In the present study, five proteins (lamin A, vimentin, tubulin-β chain, ANXA6, and SERPINC1) were reported previously, [22] and thirteen proteins were identified for the first time being differentially expressed in PCOS as compared to the normal ovaries. All the five previously identified proteins were upregulated or downregulated in agreement with our study [16, 19, 22]. Our observation complements the previously identified PCOS proteins and assists in the better understanding of the pathophysiological mechanisms of PCOS.

The present study identified significantly (P<0.01) increased expressions of HSP90B1 and CALM1 and significantly ((P<0.05) decreased expressions of ANXA6 and TPM2 in the ovarian tissues of women with PCOS (S3 Fig). The biomolecules identified by these non-targeted approaches should be considered as candidates for future studies aiming to define specific molecular phenotypes of PCOS.

The identified differentially expressed PCOS protein, HSP90B1 is a stress-inducible chaperone protein. Its expression levels have deleterious effects on cell proliferation and survival, cell cycle progression, and apoptosis. Other studies have indicated the potentially important role of HSP90B1 in the pathogenesis of PCOS [23]. HSP90B1 is demonstrated to be involved in the growth of cancerous cells, and HSP90B1 inhibitors can reverse cancer cell growth and increase survival [24–29]. HSP90B1 is an important chaperone protein that interacts with multiple intracellular receptors and transcription factors, has a role in stabilization of client proteins from protease degradation, such as AKT, I ĸB-α, glucocorticoid, and progesterone receptors [24, 25, 30], and responsible for client protein stability and maturation after activation [31]. However, the underlying molecular mechanisms of HSP90B1 on ovarian cell function remain unknown. The findings of the present study imply that HSP90B1 may have a role in promoting cell proliferation in PCOS pathogenesis. Increased granulosa cell proliferation is associated with PCOS. Granulosa cells exhibit higher proliferation rate in the ovaries of anovulatory PCOS women as compared to those with regular ovulatory function. Growth factors and stress hormones are potent inducers for cancer cell proliferation and survival, which explains why patients with PCOS are at an increased risk of endometrial cancer [11, 12]. Therefore, further studies on the downstream signaling pathways of HSP90B1 will improve our future understanding of the role and molecular mechanisms of HSP90B1 in PCOS-related cancer genesis such as endometrial cancer.

Our study also pinpointed differential expression of an intriguing protein, PGRMC1, a known progesterone regulator. We confirmed that PGRMC1 protein levels were increased in patients with PCOS compared with the controls (S3 Fig and S4 Fig). PGRMC1, approximately 22 kDa, was first described in 1998 [32] as a putative progesterone-binding membrane receptor [33]. Also, PGRMC1 is expressed in human ovarian granulosa and luteal cells [34, 35], and its expression level and subcellular localization is regulated by gonadotropins [36]. The present study is the first to identify PGRMC1expression in human ovaries (S3 Fig and S4 Fig). PGRMC1 is speculated to enhance steroidogenesis by increasing cholesterol synthesis and function via a protein kinase G–dependent mechanism and interaction with the Serpine 1 mRNA binding protein [37]. PGRMC1 could also activate cytochrome P450 [38], which plays an essential role in steroid synthesis. PGRMC1 might be linked to the hyperandrogenism mechanism in PCOS. PGRMC1 overexpression in cultured cells increases progesterone hydroxylation by cytochrome P450 Cyp21, which suggests an intracellular downstream effect of PGRMC1 [39]. In PCOS ovaries, the growth of early antral follicles is typically arrested at the 5- to 10-mm stage, resulting in ovaries with multiple follicular structures <10 mm in diameter with ovarian stroma of increased volume and density. Whether PGRMC1 plays a pivotal role in the pathogenesis of excess androgen requires further elucidation.

This is the first report to demonstrate differential identification of 14-3-3ε protein in PCOS ovaries. Expression of 14-3-3ε is linked with insufficient insulin secretion and reduced β-cell mass [40]. The 14-3-3γ dimers and exportin-1 (chromosomal region maintenance-1)-dependent nuclear export binds to phosphorylated factor forkhead box protein O1 (FOXO1) by protein kinase B [41, 42]. The 14-3-3ζ dimerization is regulated by post-translational changes. Protein kinase C δ phosphorylates 14-3-3ζ at S58, which impairs chaperone dimerization and binding to target proteins like Bcl-2-associated X protein (BAX) and Bcl-2-associated death promoter (BAD) [42–44]. In β cells, 14-3-3ζ is highly expressed and sequesters the pro-apoptotic proteins BAD and BAX in the cytosol. Accordingly, reduced 14-3-3ζ levels induce mitochondrial-linked β-cell death [45]. Gerst et al. suggested that nuclear 14-3-3ζ binds to P-FOXO1, thereby promoting nuclear extrusion of the transcription factor [40]. This additional role underlines the importance of 14-3-3ζ for β-cell function [45]. In another study, Neukamm et al. provided clear evidence that insulin receptor substrate 2 (IRS2) and 14-3-3 are binding partners under increased cyclic adenosine monophosphate (cAMP) level conditions. The binding is at least partially mediated by protein kinase A–dependent phosphorylation of IRS2 on serine 1137/1138 and enhances IRS2 protein stability. The increased IRS2 protein stability upon elevated cAMP levels provides an additional mechanism of cAMP-induced IRS2 mRNA and subsequent protein expression to ensure sufficient amounts of IRS2 protein [46]. An increasing number of studies have shown the importance of the 14-3-3 proteins in controlling cellular processes like the cell cycle, cell growth, gene transcription, and apoptosis, and are tightly linked to the pathophysiological mechanism of insulin resistance. The present study was the first to indicate that 14-3-3ζ is up-regulated in PCOS compared to control ovaries. The 14-3-3ζ protein may represent a novel target that is extremely relevant with the PCOS-related hyperinsulinemia and might be exploited in the future to develop novel therapeutics.

However, the present study suffers some limitations. The number of samples from women with PCOS was limited because of the scarcity of available patients; study with more number of samples would improve the brevity of the findings. Also, the shortage of the ovarian samples imposed limits on the IHC analysis that might have a confounding influence on the estimation of protein expression levels. Counterstaining for markers to define the cell types and correlate the protein staining to increased androgen production or cell function might enhance clarity and can be considered in future studies. The groups of patients recruited in the study were not evidently examined for hyperinsulinemia. In addition, the PCOS subjects selected had a previous history of clomiphene treatment, which might exert a confounding effect on the expression of the proteins.

The present study identified biologically relevant differentially expressed proteins in PCOS and normal ovaries, as they are involved in cellular proliferation and survival and tightly associated with the pathological mechanism of hyperandrogenism and hyperinsulinism. The identified differentially expressed biomolecules such as PGRMC1 and 14-3-3ζ might be novel proteins that are closely associated with the pathogenesis of hyperinsulinism and further hyperandrogenism of PCOS. The findings provide an important insight towards a better understanding of the dysregulated pathways in PCOS and help in developing novel therapeutic intervention strategies.

## Supporting Information

S1 Fig Proteins differentially expressed in PCOS and normal ovaries. The proteins were analyzed by two-dimensional DIGE.
(TIF)

S2 Fig Eighteen differentially expressed proteins. The MALDI-TOF MS analysis spots are labeled in red boxes that show differences between the samples from the PCOS patients and controls.
(TIF)

S3 Fig Western blot (WB) analysis of the differentially expressed proteins. In ovarian tissues of patients with PCOS the expression of PGRMC1, RBP1, HSP90B1, and CALM1 was increased, whereas that of ANXA6 and TPM2 was decreased. The expression of HSP90B1, CALM1, ANXA6, and TPM2 proteins from ovarian biopsies of control and PCOS was analyzed by WB. (A) Representative bands from the control (n = 10) and PCOS (n = 10) analyzed samples. GAPDH was used as an internal control. (B) The mean ratio, control (n = 10) and PCOS (n = 10) group, of the target protein to GAPDH ± standard error (SE, each of the samples was analyzed in three independent experiments). * P<0.05, ** P<0.01 vs. control group. The level of protein expression was quantitatively analyzed by densitometric analysis for each of the twenty samples.
(TIF)

S4 Fig Immunohistochemistry (IHC) of the protein expression in ovary tissue. PGRMC1, RBP1, HSP90B1, CALM1, ANXA6, and TPM2 expression was investigated in PCOS (n = 10) and control (n = 10) ovaries. (A) Representative IHC image from all of the twenty analyzed samples. The identified proteins were analyzed by IHC, and the stained sections were developed using DAB. (B) The detected protein expression for the each of the PCOS (n = 10) and control (n = 10) samples was quantitatively analyzed by cell counting, and the data are presented as mean ratio of cell counts of positive cells to nuclei ± SE (n = 10). * P<0.05, ** P<0.01 vs. control group.
(TIF)

S1 Table Samples Arrangement for a Triplicate 2D-DIGE Experiment. (DOC)

S2 Table Specimen collection in control group. (DOC)
