# Supplementary figures and images for: Proteomic Profiling for Identification of Novel Biomarkers Differentially Expressed in Human Ovaries from Polycystic Ovary Syndrome Patients
Source: PLoS One. 2016 Nov 15;11(11):e0164538. doi: 10.1371/journal.pone.0164538 (PMC5112797; doi:10.1371/journal.pone.0164538)

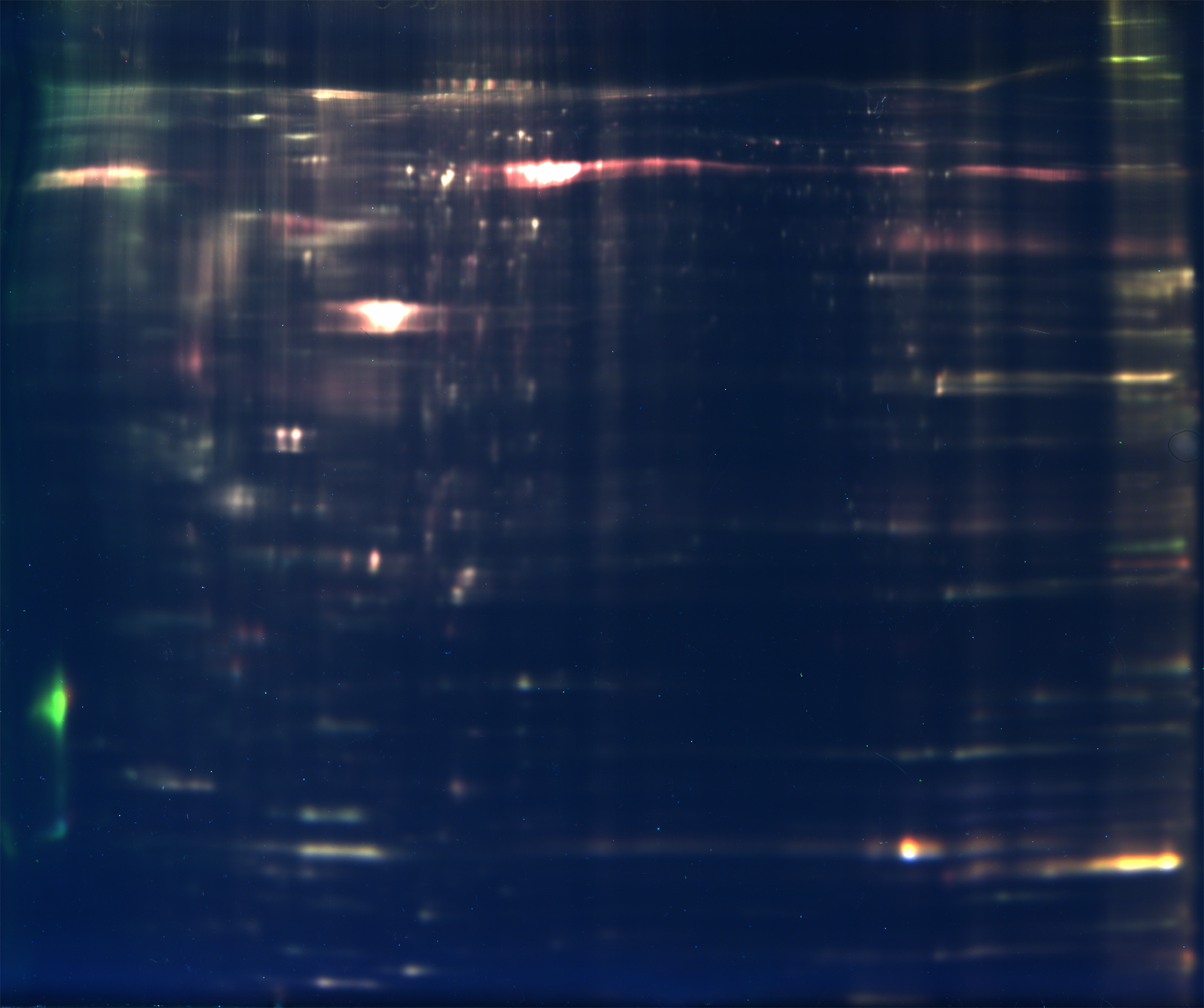

Supplement: S1 Fig — The proteins were analyzed by two-dimensional DIGE. (TIF) [file pone.0164538.s001.tif]

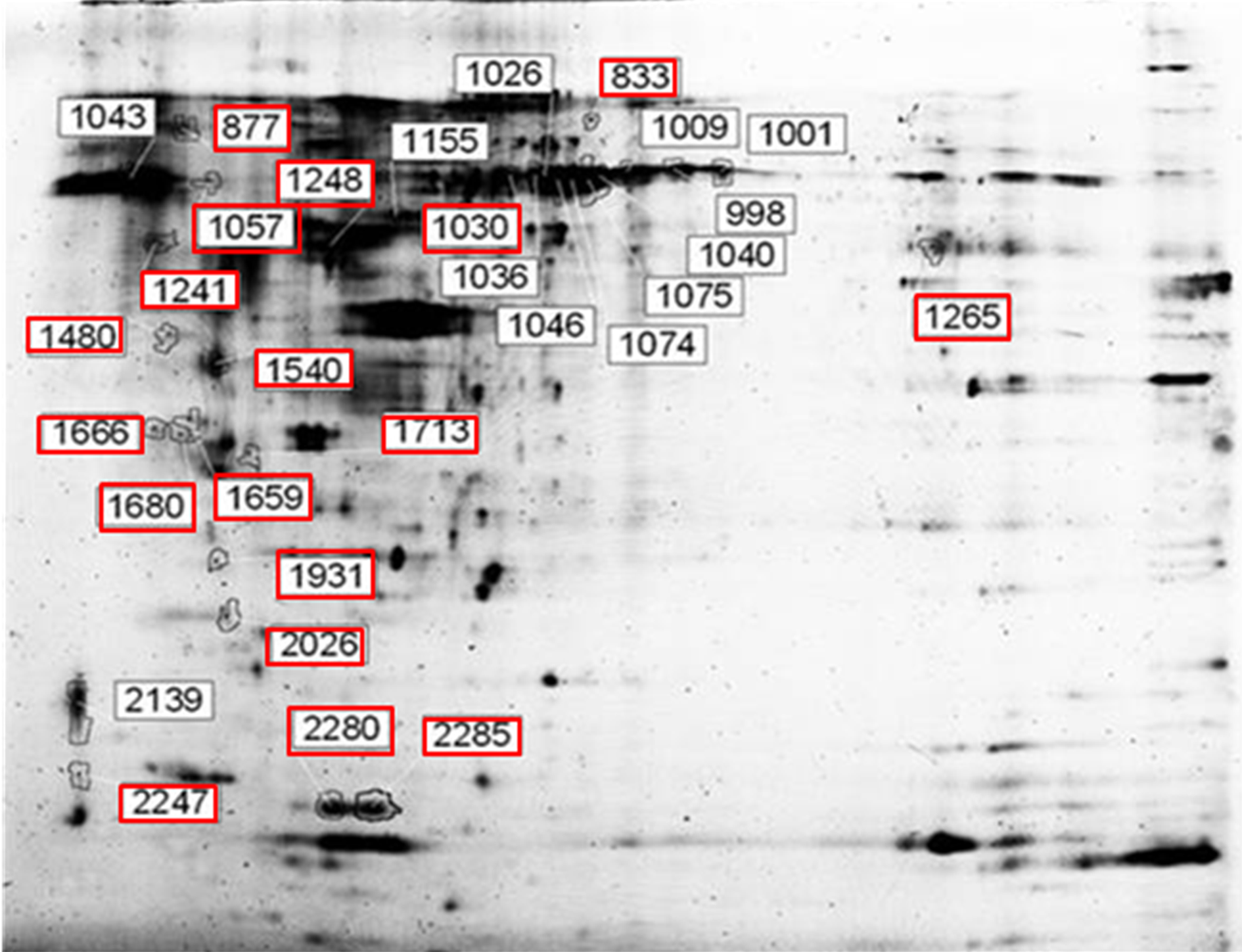

Supplement: S2 Fig — The MALDI-TOF MS analysis spots are labeled in red boxes that show differences between the samples from the PCOS patients and controls. (TIF) [file pone.0164538.s002.tif]

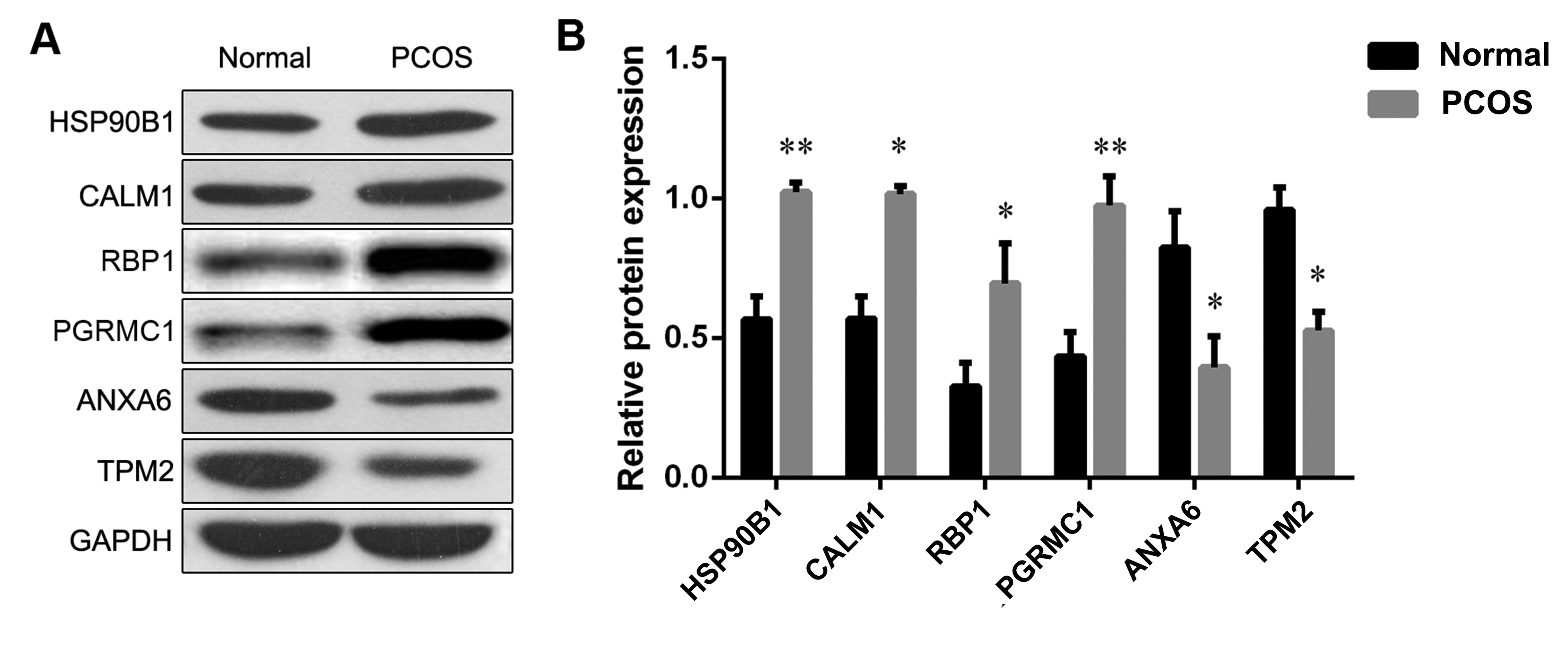

Supplement: S3 Fig — In ovarian tissues of patients with PCOS the expression of PGRMC1, RBP1, HSP90B1, and CALM1 was increased, whereas that of ANXA6 and TPM2 was decreased. The expression of HSP90B1, CALM1, ANXA6, and TPM2 proteins from ovarian biopsies of control and PCOS was analyzed by WB. (A) Representative bands from the control (n = 10) and PCOS (n = 10) analyzed samples. GAPDH was used as an internal control. (B) The mean ratio, control (n = 10) and PCOS (n = 10) group, of the target protein to GAPDH ± standard error (SE, each of the samples was analyzed in three independent experiments). * P<0.05, ** P<0.01 vs. control group. The level of protein expression was quantitatively analyzed by densitometric analysis for each of the twenty samples. (TIF) [file pone.0164538.s003.tif]

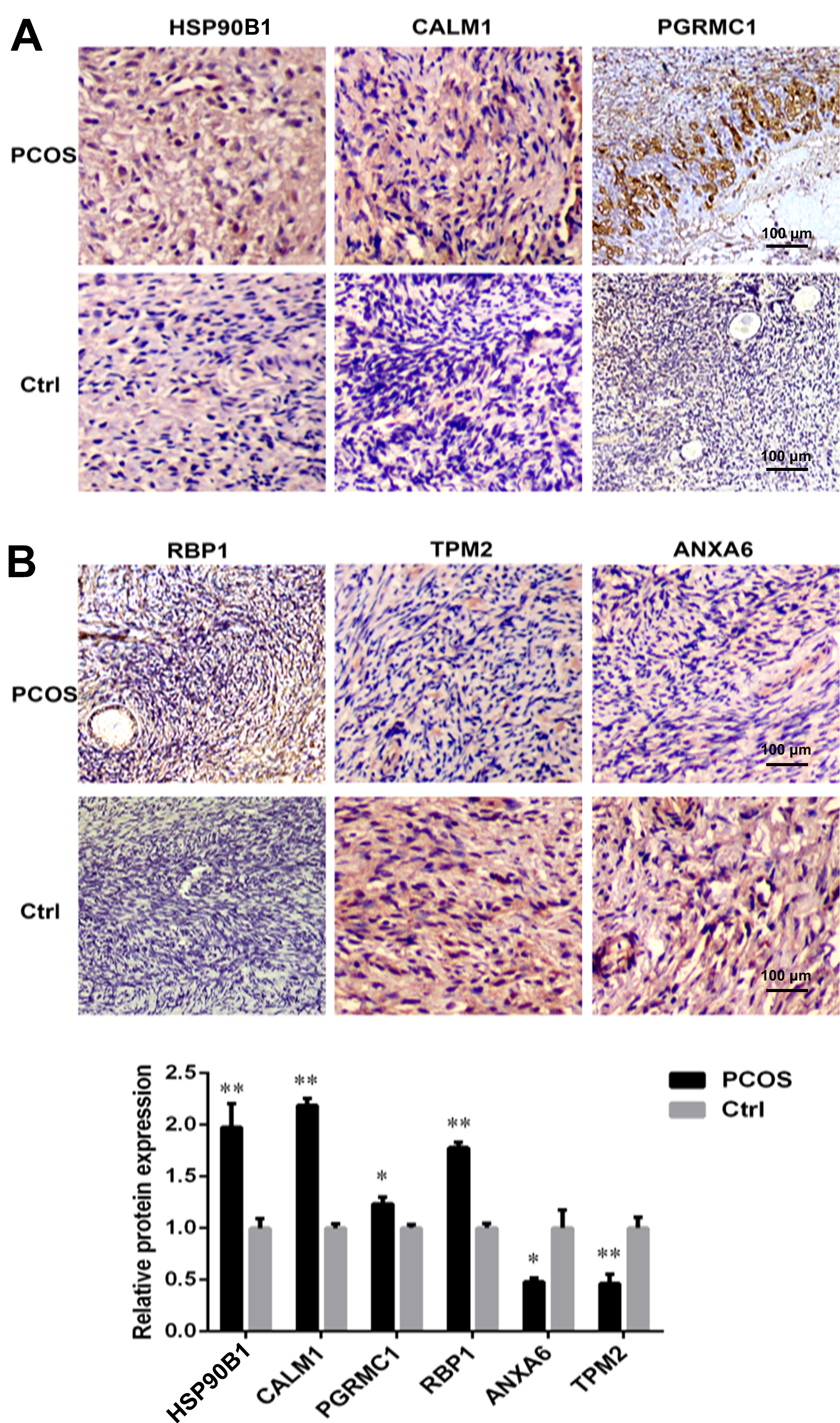

Supplement: S4 Fig — PGRMC1, RBP1, HSP90B1, CALM1, ANXA6, and TPM2 expression was investigated in PCOS (n = 10) and control (n = 10) ovaries. (A) Representative IHC image from all of the twenty analyzed samples. The identified proteins were analyzed by IHC, and the stained sections were developed using DAB. (B) The detected protein expression for the each of the PCOS (n = 10) and control (n = 10) samples was quantitatively analyzed by cell counting, and the data are presented as mean ratio of cell counts of positive cells to nuclei ± SE (n = 10). * P<0.05, ** P<0.01 vs. control group. (TIF) [file pone.0164538.s004.tif]
