# Supplementary material for: Proteomic Profiling for Identification of Novel Biomarkers Differentially Expressed in Human Ovaries from Polycystic Ovary Syndrome Patients
Source: PLoS One. 2016 Nov 15;11(11):e0164538. doi: 10.1371/journal.pone.0164538 (PMC5112797; doi:10.1371/journal.pone.0164538)
Supplement: S1 Table — (DOC) [file pone.0164538.s005.doc]

**S1 Table**. **Samples Arrangement for a Triplicate 2D-DIGE Experiment.**

| Gel No. | Cy2 (50 μg) | Cy3 (50 μg) | Cy5 (50 μg) |
| --- | --- | --- | --- |
| Gel 1 | Pool | Control 1 | PCOS 1 |
| Gel 2 | Pool | PCOS 2 | Control 2 |
| Gel 3 | Pool | Control 3 | PCOS 3 |
