# Supplementary material for: Proteomic Profiling for Identification of Novel Biomarkers Differentially Expressed in Human Ovaries from Polycystic Ovary Syndrome Patients
Source: PLoS One. 2016 Nov 15;11(11):e0164538. doi: 10.1371/journal.pone.0164538 (PMC5112797; doi:10.1371/journal.pone.0164538)
Supplement: S2 Table — (DOC) [file pone.0164538.s006.doc]

S2 Table. Specimen collection in control group

| Control | Procedure performed | Last menstrual period | Collecting Date |
| --- | --- | --- | --- |
| 1 | Right ovarian teratoma resection under laparoscope | 2013-11-3 | 2013-11-15 |
| 2 | Left ovarian cyst resection under laparoscope | 2013-11-7 | 2013-11-19 |
| 3 | Left ovarian teratoma resection under laparoscope | 2013-11-6 | 2013-11-20 |
| 4 | Right ovarian cyst resection under laparoscope | 2013-10-31 | 2013-11-19 |
| 5 | Left adnexa resection under laparoscope | 2013-8-8 | 2013-8-29 |
| 6 | Right ovarian teratoma resection under laparoscope | 2013-11-15 | 2013-11-27 |
| 7 | Right ovarian cyst resection under laparoscope | 2013-11-19 | 2013-12-3 |
| 8 | Bilateral Messoalpinx cyst resection under laparoscope, removal of IUD | 2013-11-29 | 2013-12-16 |
| 9 | Left ovarian cysts resection under laparoscope | 2013-12-6 | 2013-12-16 |
| 10 | Left ovarian theca lutein ovarian cyst resection under laparoscope | 2014-1-4 | 2014-1-13 |
